# Role of the cGAS-STING pathway in radiotherapy for non-small cell lung cancer

**DOI:** 10.1186/s13014-023-02335-z

**Published:** 2023-09-04

**Authors:** Chunsheng Yang, Yan Liang, Ning Liu, Meili Sun

**Affiliations:** 1 Department of Oncology, Central Hospital Affiliated to Shandong First Medical University, Jinan City, China; 2 Department of Radiation, The Second Affiliated Hospital of Xingtai Medical College, Xing Tai Shi, China

**Keywords:** Radiation, Non-small cell lung cancer, cGAS–STING signaling, Lung injury, Interferon, Antitumor immunity

## Abstract

One of the most important therapeutic interventions for non-small cell lung cancer is radiotherapy. Ionizing radiation (IR) is classified by traditional radiobiology principles as a direct cytocidal therapeutic agent against cancer, although there is growing recognition of other antitumor immunological responses induced by this modality. The most effective therapeutic combinations to harness radiation-generated antitumor immunity and enhance treatment results for malignancies resistant to existing radiotherapy regimens could be determined by a more sophisticated understanding of the immunological pathways created by radiation. Innate immune signaling is triggered by the activation of cGAS-STING, and this promotes adaptive immune responses to help fight cancer. This identifies a molecular mechanism radiation can use to trigger antitumor immune responses by bridging the DNA-damaging ability of IR with the activation of CD8 + cytotoxic T cell-mediated killing of tumors. We also discuss radiotherapy-related parameters that affect cGAS-STING signaling, negative consequences of cGAS-STING activation, and intriguing treatment options being tested in conjunction with IR to support immune activation by activating STING-signaling. Improved therapeutic outcomes will result from a better understanding of how IR promotes cGAS-STING signaling in immune-based treatment regimens that maximize radiotherapy’s anticancer effectiveness.

## Introduction

Lung cancer is one of the most common cancers and the leading cause of cancer-related deaths worldwide [1]. Among the common lung cancer types 85% of clinical cases are non-small cell lung cancer (NSCLC) [2, 3],which has a low overall cure rate and survival rate [4, 5]. Radiotherapy (RT), as a traditional treatment, is applied to more than 50% of patients with malignant tumors, and its principle of action is to directly cause fatal DNA damage in irradiated cells, or indirectly induce DNA damage by producing reactive oxygen species [6]. RT induces DNA damage, resulting in the formation of micronuclei containing chromatin and dsDNA in the cytosol, and the nuclear envelope of micronuclei is easily ruptured due to the lack of a stable nuclear lamina, exposing dsDNA to the cytoplasm and becoming the trigger point of the innate immune response, exerting anti-tumor effects [7]. Radiation therapy plays an important role in the treatment of non-small cell lung cancer, and inevitable lung tissue will be irradiated at a certain dose during the treatment, causing different degrees of radiation-induced lung injury, with an incidence of about 5–20%, and reducing the local control rate of the tumor to a certain extent, becoming an important limiting factor in the dose of radiotherapy [8, 9].

Cyclic guanosine monophosphate-adenosine monophosphate synthase (cGAS)/interferon stimulating factors (STING) signaling pathway, as an important part of the innate immune system, plays an important role in maintaining the homeostasis of the body. Blocking the cGAS-STING pathway inhibited inflammatory response and reduced tissue damage, while activating the cGAS-STING pathway promoted antiviral and anti-tumor effects. Understanding the regulatory mechanism of cGAS-STING pathway links can use existing drugs or new drugs developed to intervene in the cGAS-STING pathway and provide new ideas and methods for the treatment of clinically relevant diseases. The purpose of this review is to discuss the immune activation mechanism of cGAS-STING in lung cancer radiotherapy and the mechanism of lung injury and to discuss new therapeutic methods.

## Structure and signal transduction of the cGAS/STING pathway

**Fig. 1:**
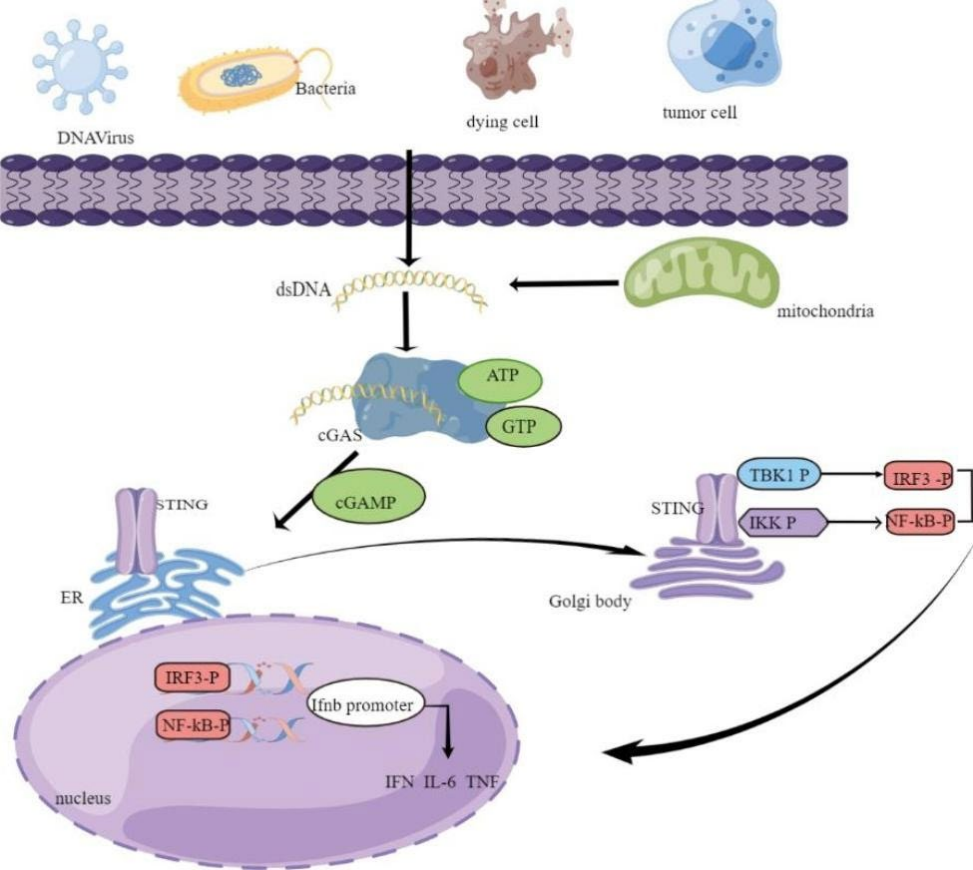
The cGAS-STING DNA sensing signaling pathway

The cGAS-STING signaling pathway is an important cytosolic DNA sensing pathway in vivo, which induces the expression of type I IFNs and affects the immune response of the body, and plays an important role in regulating pathogen infection, tumor immunity,and autoimmune diseases. cGAS has a highly conserved nucleotidyltransferase domain and can detect double-strands DNA (dsDNA) released into the cytosol. The Binding of cGAS and dsDNA is length-dependent and independent of DNA sequences [10]. dsDNA binds to cGAS as a dimer in a 2:2 manner, causing a conformational change in the active site of cGAS [11], which in turn promotes ATP and GTP synthesis of the second messenger cyclic guanylin acid-adenylate (cGAMP) [12]. Synthetic cGAMP binds to STING molecules localized as homodimers on the ER [13], resulting in conformational changes in STING to form STING tetramers and higher-order oligomers. This conformational change induces activation of STING, which shifts to a more closed conformation and translocates to the Golgi through the ER-Golgi intermediate compartment [14]. Structural studies have shown that two cysteine residues on STING protein are palmitoylated in the Golgi apparatus and recruit and activate TANK binding kinase-1 (TBK1) in the Golgi apparatus [15, 16]. Activation of TBK1 in turn transphosphorylates the C-terminal domain of STING, thereby recruiting interferon regulatory factor 3 (IRF3) [17]. Phosphorylated IRF3 dimerizes and translocates into the nucleus to activate transcription of genes encoding type I IFNs 1 7. STING can also simultaneously activate IκB kinases (IKK) to mediate the transcription of interleukin-6 (IL-6), tumor necrosis factor (TNF), and inflammatory cytokines such as type I IFN activated by nuclear factor kappa-B (NF-κB) [18]. It plays an immunoregulatory role and affects the body’s viral defense, inflammation, and cancer treatment.(Fig. 1).

## Antitumor immunity in the cGAS/STING pathway versus radiotherapy 

DNA, as the blueprint of life, is regulated by DNA damage response or DNA damage repair system (DDR) to ensure the integrity of the genome. When DNA is damaged, the DDR pathway causes cell cycle arrest and repairs DNA, ensuring that cell cycle cycles occur orderly; however, when damage cannot be effectively repaired, cells enter senescence; if DNA is severely damaged, it directly leads to apoptosis [19]. Radiotherapy (RT) is used as a traditional treatment in more than 50% of patients with malignant tumors, and its principle of action is to directly cause fatal DNA damage in irradiated cells, or indirectly induce DNA damage by producing reactive oxygen species [6]; its types of induced DNA damage include base mutations, single-strand breaks (SSBs), and double-strand breaks (DSBs) [20]. Radiation induction regulates the tumor microenvironment and promotes the recruitment and infiltration of immune cells. Radiation therapy (RT) can cause DNA damage in tumor cells [21], leading to chromosomal instability (CIN). CIN is a characteristic feature of cancer and it will induce micronuclei formation [22, 23].Human mtDNA’s nucleotide sequence was discovered in 1981 to be a 16 569 bp double-stranded circular molecule that codes for 37 genes that can express a number of important proteins involved in oxidative phosphorylation and essential for cellular energy metabolism [24]. The permeability of the inner and outer mitochondrial membranes varies when cancer cells experience oxidative stress and mitochondrial malfunction, and mitochondrial DNA can exit the mitochondrial outer membrane into the cytosol by mitochondrial membrane permeability transition holes or BAX/BAK-dependent permeabilization of the outer mitochondrial membrane. Inflammation is brought on by mtDNA translocation into the cytoplasm, cGAS recognition and binding, and subsequent activation of downstream immune pathways [25]. In a model of cisplatin-induced acute kidney injury, cisplatin caused mtDNA leakage into the cytoplasm through the mitochondrial outer membrane BAX/BAK pore, activated the cGAS-STING signaling pathway, and resulted in inflammation and acute kidney injury. By inhibiting STING, however, the cisplatin-induced renal inflammation could be reduced [26]. dsDNA from micronuclei as well as DNA damage induction will be recognized by the cytoplasmic DNA sensor cGAS, thereby activating the cGAS-STING signaling pathway, shaping innate immunity in a type I IFN-dependent manner, promoting adaptive immune responses, and thus effectively anti-tumor [27, 28].

**Fig. 2:**
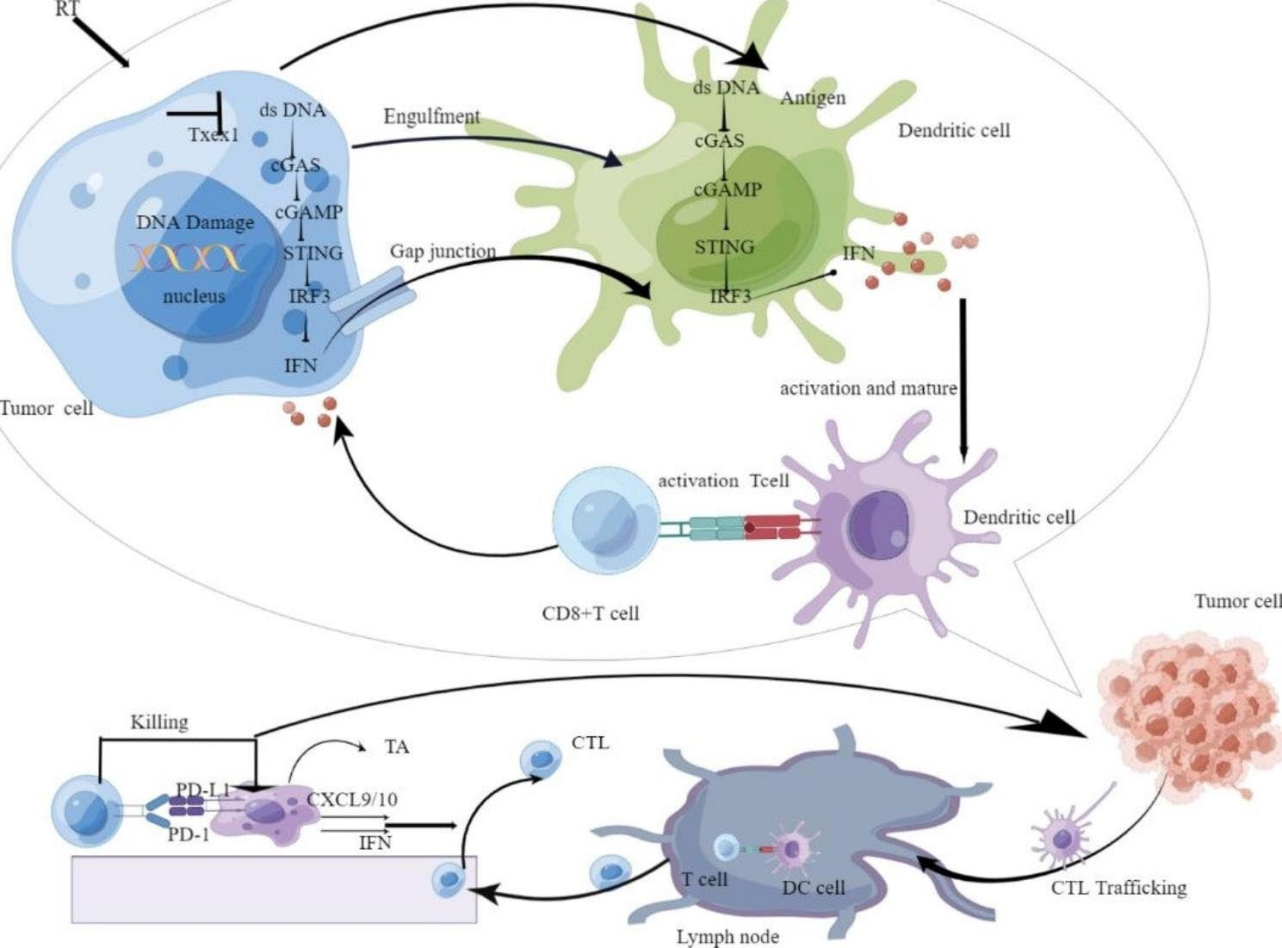
The cGAS–cGAMP–STING pathway detects cytoplasmic DNA after Ionizing radiation and activate type I IFNs and other cytokines CTL, cytotoxic T lymphocytes; CXCL9, chemokine (C-X-C motif) ligand 9; IFN, interferon; PD-1/PD-L1, anti-programmed death-1/programmed death-ligand 1; TA, tumor antigen;

Cancer cells are irradiated and exposed to tumor-specific antigens, which make them visible for immune surveillance and promote the activation of cytotoxic T cells. In the absence of STING in the host, the amount of IFN-β induced in the tumor decreased after irradiation. Consistent with the immunogenicity of IFNs, the radiation effect was attenuated in STING deficient mice compared with controls, indicating that STING dependent cytosolic DNA sensing plays a critical role in the radiation effect in vivo. Generally, tumor cells escape immune response through the inactivation of the STING signaling pathway. When the cGAS-STING signaling pathway in tumor cells is activated, it induces expression of cytokines such as IL-6, TNF, and type I IFN, leading to tumor cell death or apoptosis, releases dsDNA, and other tumor-derived antigens, activates dendritic cells (DCs), and then initiates anti-tumor immunity. In the tumor microenvironment, tumor-derived DNA can be taken up by DCs through unknown mechanisms, further triggering stronger adaptive anti-tumor immune response [29]. Tumor cells enhanced cross-antigen presentation by DCs after irradiation, whereas DCs lacking STING was unable to cross-infect primary CD + 8 T cells [30]. Compared with irradiation alone, cGAMP combined with irradiation effectively reduced tumor burden in vivo, indicating that cGAMP treatment enhanced radiotherapy efficacy. DNA produced by tumor cells after radiotherapy promotes the anti-tumor effect of radiotherapy by triggering the cGAS/STING pathway to enhance T cell responses in mice to induce anticancer immunity in a STINE-dependent manner [30]. However, whether radiotherapy can induce cGAS-STING mediated anti-tumor effect is related to the applied radiation dose, too high dose will affect the survival of immune cells in the tumor microenvironment, but also up-regulate the negative regulators of the DNA sensing pathway, inhibit cGAS-STING pathway activation, and then promote immunosuppression, while lower dose can effectively activate the cGAS/STING pathway. Vanpouille-Box et al. found that Trex1 is a DNA exonuclease that acts as an upstream regulator of RT-driven anti-tumor immunity, and the DNA exonuclease Trex1 is produced in different cancer cells after irradiation at doses of 12 to 18 Gy, mainly by degrading cytosolic DNA, resulting in a decrease in dsDNA, a ligand required to activate cGAS/STING, and inhibiting anti-tumor immune effects [31].Repeated irradiation at doses that do not induce Trex1 promotes IFN-β production, thereby recruiting and activating Batf3-dependent DCs. This effect is essential for priming of CD + 8 T cells that mediate systemic tumor rejection. Radiotherapy leads to dsDNA accumulation in cancer cells when Trex1 is not activated type I IFNs are activated through the cGAS/STING pathway, downstream recruitment of DCs, and activation of CD + 8 T cells or anti-PD-1 antibodies to initiate tumor rejection. In tumors treated with irradiation doses exceeding the Trex1 induction threshold, clearance of dsDNA from cancer cell sol hinders IFN-β release in cancer cells, resulting in a reduced recruitment of DCs and insufficient activation of CD + 8 T cells leading to diminished local tumor regression [32]. Jason J. found unfavorable local tumor response was linked to overexpression of the DNase III (TREX1) exonuclease [33].RT acts as an inducer of antitumor immune responses, in part, depending on the type I IFN secretion caused by activation of the cGAS/STING signaling pathway and the interaction between immune cells. Exosomes are membrane microvesicles that range in size from 30 to 100 nm and are released by all types of cells. They offer a highly developed method of local and distant intercellular communication [34]. Julie M showed that irradiated mouse breast cancer cells (RT-TEX) create tumor-derived exosomes (TEX) that transmit dsDNA to DCs and cause them to upregulate costimulatory molecules and STING-dependent IFN-I activation [35].Therefore, exploring the molecular mechanisms affecting the activation of this pathway will provide new targets for enhancing RT-induced anti-tumor immunity.(Fig. 2).

## cGAS/STING pathway and radiation-induced lung injury

Radiation therapy is one of the important treatment methods for thoracic malignant tumors. During the treatment, inevitable lung tissues will be irradiated with a certain dose [36], which causes different degrees of radiation-induced lung injury, with early manifestations of radiation pneumonitis and radiation-induced pulmonary fibrosis, thus making it difficult for the chest irradiation dose routinely used in clinical practice to reach the dose required for radical resection of tumors, and it is also difficult to further improve the local tumor control rate in patients with thoracic tumors, especially inoperable non-small cell lung cancer. Radiation-induced lung injury occurs in 13–37% of patients receiving radiotherapy for lung cancer [37], and with the substantial progress in the technology level and equipment of radiotherapy in recent years, the incidence of R ILI, although decreasing, is still higher than 15% [38, 39], and the mortality rate is as high as 4% [40]. It is an important dose-limiting factor in thoracic radiotherapy and affects the quality of life of patients.

High doses of ionizing radiation are well-known to cause DNA double-strand breaks [41]. DNA damage leads to oxidative stress, vascular damage, and inflammation. Pneumonia develops within hours or days of high-dose irradiation and is associated with increased capillary permeability and accumulation of inflammatory cells in the lung [9, 42]. The recruited inflammatory cells secrete profibrotic cytokines to activate resident fibroblasts, ultimately leading to excessive collagen production and deposition in the interstitial space of the lung. Some major signaling pathways have been identified that are associated with the amplification of inflammatory cytokine cascades, with NF-κB playing an important role in the regulation of gene expression of various inflammatory cytokines [43]. NF-κB is an important transcription factor widely present in eukaryotic cells, which can participate in the transcriptional regulation of a variety of genes and is closely related to important physiological and pathological processes such as immune response, inflammatory response, as well as cell proliferation, differentiation, and apoptosis [44]. NF -κB binds to its inhibitor protein IκB at rest and is inactive [45]. When cells are stimulated by external stimuli, such as oxidative stress, infection, or physicochemical stimuli, they can activate the transcriptional activity of NF-κB and promote the acidification and degradation of IκB, thereby releasing NF-κB into the nucleus and binding to the κB site of the target gene, rapidly inducing target gene transcription and up-regulating the expression of various biomacromolecules, including inflammatory cytokines, chemokines, and apoptosis-related factors. Among them, inflammatory factors such as TNF -α and I L-1β can also up-regulate NF-κB transcriptional activity, thus forming positive feedback and continuously expanding the stimulation signal [46].The continuous expression and accumulation of these inflammatory factors can cause damage to tissue cells. Studies have shown that nuclear factor-κB inflammation-related pathways and cytokines mainly mediate inflammatory responses such as tumor necrosis factor TNF -α, interleukin − 1, and interleukin − 6 play an important role in the initiation and promotion of radiation-induced lung injury. NF-κB is continuously activated throughout radiation-induced lung injury, which may be one of the causes of persistent chronic inflammation [47]. NF-κB is a major switch that regulates the body’s inflammatory response. When the body is irradiated, intracellular DNA damages and activates the cGAS/STING pathway, phosphorylates NF-κB to inhibit protein kinase, so that NF-κB inhibitory protein IκB is ubiquitinated and degraded, thus activating NF-κB into the nucleus to initiate the release of a variety of inflammatory factors. Activation of inflammatory factors in the lung stimulates the phenotypic transformation of lung epithelial cells into intercellular cells, producing large amounts of extracellular matrix proteins that accumulate and deposit in the pulmonary interstitium and promote the formation of pulmonary fibrosis [48]. Therefore, reducing lung fibrosis and lung inflammation effectively protects against RILI. Because NF-κB factor is a heterodimer composed of two peptides, p 50 and p65, and only the C -terminus of p 65 protein contains a transcriptional activation region that can act directly on targeted genes to activate transcription, changes in NF-κB activity can currently be evaluated by detecting the expression of p 65. Related studies have shown that 3,3 ′-diindolylmethane (DIM) can inhibit the NF-κB pathway, and in mouse models, DIM can effectively ameliorate RILI-induced pulmonary fibrosis and lung inflammation while reducing the number of inflammatory cells in BALF as shown by H E staining HE staining and Masson staining [48]. Savita et al. showed that Quercetin-3-Rutinoside provides radioprotection to the lung by regulating NF-κB/TGF-β1 signaling, scavenging free radicals, preventing perivascular invasion, and prolonging the inflammatory cascade, which may otherwise lead to chronic radiation fibrosis. Inhibition of the cGAS/STING/NF-κB pathway is therefore a potential target for the treatment of radiation-induced lung injury.

## cGAS/STING pathway combined with radiotherapy immunotherapy

Immune checkpoint inhibitors mainly include PD-1/PD-L1 antibody and CTLA-4 antibody. Theoretically, ICI therapy can re-edit the tumor immune microenvironment to induce tumor regression, and the activity of PD-1/PD-L1 immune checkpoint inhibitors varies in different cancers, and many studies have demonstrated that they confer resistance in multiple cancer types. The effectiveness of immune checkpoint inhibitors depends on innate antitumor immunity, particularly the production of tumor antigens and tumor-specific cytotoxic T cells (CTLs). However, most cancer patients remain unresponsive to checkpoint inhibitor therapy, most of which are due to their inability to mount sufficient antitumor immunity.

Numerous studies have shown weak responses to multiple immune checkpoints in STING null mice, including PD-1, PD-L1, CTLA4, and CD47 [47]. STING activators are ideal sensitizers for enhancing anti-PD-1/PD-L1 therapy, and STING agonists can significantly enhance cytotoxic T cell infiltration in tumor cells [49]. Immune checkpoints negatively regulate T cells in the immune system, and immune checkpoint blockade therapy has shown good efficacy in tumors, while the STING signaling pathway is essential for the anti-tumor effect of immune checkpoint blockade therapy in mice. In wild-type mouse melanoma models, intramuscular injection of cGAMP away from the tumor site significantly enhanced the therapeutic effect of immune checkpoint blockade, and the combination of cGAMP and PD-L1 antibody effectively inhibited tumor growth in B16 melanoma mice45. When cGAS, STING is absent, responses to immunotherapies such as immunosuppressive molecule blockade are weak, and the combination of STING agonists and PD-1 blockers shows stronger antitumor efficacy, and when combined, tumor models that do not respond well to immune checkpoint blockade become sensitized [50]. Radiotherapy has gained broad public consensus as an adjuvant to activate immune responses. Jason R. Baird et al. demonstrated synergistic local and distant tumor control when radiation therapy was combined with a novel STING ligand in a mouse model of pancreatic cancer [51]. Radiation therapy can act synergistically with PD-1 inhibitors to enhance their respective antitumor activity, particularly in patients with advanced tumors [52].

NF-κB control plays a crucial role in immune-inflammatory responses, tumorigenesis, and development/radioresistance. Inhibition of the canonical NF-κB pathway has been shown to attenuate radiation efficacy, whereas non-canonical NF-κB deficiency promotes radiation-induced antitumor immunity [53]. Mechanistic studies have shown that non-canonical NF-κB signaling in dendritic cell DCs is associated with activation of the DNA-reception-mediated STING pathway [53]. It has been shown that modulation of type I interferons by application of the NF-κB2 competitive inhibitor S N52 facilitated the therapeutic effect of IR, but tumors receiving combination therapy were not completely eliminated. Sustained IFN-I signaling induces immunosuppressive mechanisms, including PD-L1 expression on DCs and other myeloid cells and PD-1 expression on T cells, which leads to CD8 + T cell depletion [54, 55]. Tumor-bearing mice were treated with anti-PD-L1 agents following SN52 and IR treatment. PD-L1 blockade enhanced the therapeutic effect of the SN52 + IR combination and led to tumor rejection. To investigate whether this combined treatment could generate long-lasting protective T cell immunity, tumor-free mice were rechallenged with high-dose MC38 tumor cells on the contralateral side. A few weeks later, no palpable tumors were detected in treated mice. These findings reveal that combining IR with manipulation of the STING-IFN pathway and immune checkpoint inhibition can better activate innate immunity and reduce immunosuppression, and may provide a novel approach for the treatment of tumors, that is, inhibition of non-canonical NF-kB plus antiPD-L1 antibody drugs [55].

## Concsulion

Activation of the cGAS-STING pathway in lung cancer radiotherapy has shown great antitumor potential by promoting the secretion of type I IFNs, significantly increasing the infiltration of CD + 8 T cells and effectively stimulating anti-tumor immune responses in the body. When exosomes deliver dsDNA from radiation-damaged cancer cells to DCs, type I interferon (IFN-I) is activated via the cGAS/STING pathway.RT-TEX serve as transmitters of the molecular alterations that occurred in radioactively treated cancer cells. Adjuvants that activated DCs and might trigger protective antitumor T-cell responses were conveyed by RT-TEX together with tumor antigens.When the STING signaling pathway is used for anti-tumor therapy, therapeutic window and toxic side effects should be considered, and excessive activation of the pathway is associated with the occurrence of related injuries. Although the combination of radiation and immunotherapy has proven effective in preclinical studies, the future application of this combination remains challenging. Optimization of radiation dose and time and identification of potential biomarkers can further enhance the effectiveness of this unique combination, and a better understanding of how the cGAS/STING pathway mediates anti-tumor effects will contribute to the use of STING agonists in cancer therapy and design better treatment regimens.

## Data Availability

Not applicable.
